# Editorial: Microbial adaptation and metabolic reprogramming under emerging contaminants stress in engineered water/wastewater treatment ecosystems

**DOI:** 10.3389/fmicb.2026.1978908

**Published:** 2026-09-10

**Authors:** Qi Zhou, Yanchu Ke, Chao Zhang

**Affiliations:** 1 School of Energy and Environmental Engineering, Beijing Key Laboratory of Resource-oriented Treatment of Industrial Pollutants, University of Science and Technology Beijing, Beijing, China; 2 State Key Laboratory of Iron and Steel Industry Environmental Protection, Beijing, China; 3 Fujian Provincial Key Laboratory of Soil Environmental Health and Regulation, College of Resources and Environment, Fujian Agriculture and Forestry University, Fuzhou, China; 4 Guangdong Provincial Engineering Research Center of Intelligent Low-carbon Pollution Prevention and Digital Technology and Guangdong Provincial Key Laboratory of Chemical Pollution and Environmental Safety and Ministry of Education Key Laboratory of Theoretical Chemistry of Environment, School of Environment, South China Normal University, Guangzhou, China

**Keywords:** antibiotic resistance (ABR), emerging contaminants (ECs), engineered wastewater treatment, metabolic trade-offs, microbial community assembly/succession, per- and polyfluoroalkyl substances (PFAS)

Engineered water and wastewater treatment ecosystems rely fundamentally on complex and dynamic microbial communities. These communities drive the transformation of organic matter, nutrients, and diverse contaminants while maintaining the functional stability of treatment processes. However, the increasing occurrence of emerging contaminants (ECs), such as antibiotics, per- and polyfluoroalkyl substances (PFAS), microplastics, and herbicide residues, together with climate-related environmental disturbances, is creating new and persistent selective pressures on engineered microbiomes (Zhao et al., 2025). Understanding contaminant removal alone is therefore no longer sufficient. A central challenge is to determine how microorganisms sense environmental stress, reorganize their metabolism, exchange genetic information, restructure ecological interactions, and ultimately maintain or lose ecosystem functions (Peng et al., 2025).

This Research Topic, Microbial Adaptation and Metabolic Reprogramming under Emerging Contaminants Stress in Engineered Water/Wastewater Treatment Ecosystems, was established to advance this emerging perspective. The four contributions collected in this topic span multiple biological and ecological scales, ranging from molecular defense and metabolic trade-offs to microbial community assembly, horizontal gene transfer, and biological sensing. Although they address different contaminants and environmental systems, these studies converge on a common message: emerging contaminants are not merely external pollutants to be removed, but also ecological and evolutionary drivers capable of reshaping microbial systems.

A major theme emerging from this Research Topic is the importance of ecological organization in microbial adaptation. Xu et al. tackled herbicide stress (glyphosate and atrazine) from a community ecology perspective. They designed multi-trophic systems incorporating bacteria, wetland plants, and plant-microbe continuums. Their findings demonstrated that the introduction of decomposer trophic levels amplified stochastic effects on effluent quality and maintained community stability under stress through modulation of metabolic and environmental responsiveness. This work highlight that microbial adaptation cannot be understood solely by identifying individual functional taxa or evaluating contaminant removal efficiency. Instead, trophic relationships among decomposers can mediate positive interactions between microbial community assembly, ecosystem function, and system stability. The work of Xu et al. therefore provides evidence for viewing microbial adaptation as a collective ecological process. Community-level organization, rather than the response of a single dominant organism, may ultimately determine whether engineered microbiomes can maintain stable functions under long-term contaminant exposure.

Microbial adaptation also occurs through evolutionary and genetic processes. Bradshaw integrating three underexplored dimensions of mobile genetic elements (MGE)-mediated antibiotics resistance gene (ARGs) dissemination in wastewater treatment plants (WWTPs). First, they discussed how microplastics and PFAS promote horizontal gene transfer (HGT) by enhancing cell-cell contact and inducing SOS responses. Second, they addressed climate change impacts, extreme weather events can overwhelm WWTPs, introduce diverse genetic material, and shift HGT dynamics (Brown et al., 2024). Third, they raised concerns about trophic transmission, whereby MGEs and ARGs move from WWTP microbiomes to macro-organisms via food webs. This review convincingly argues that EC-driven microbial adaptation must be viewed as a coupled evolutionary-ecological process operating across scales from genes to ecosystem. Also, this perspective raises an important question for environmental biotechnology: how can engineered microbial systems simultaneously achieve efficient pollutant removal while minimizing unintended genetic and ecological risks?

A third perspective presented in this Research Topic concerns the need to better monitor the biological consequences of emerging contaminants. Gan et al. proposed a paradigm shift for PFAS detection using whole-cell bioreporters (WCBs), genetically engineered microorganisms producing detectable signals upon PFAS exposure. WCBs offer cost-effective, rapid, *in-situ* detection capabilities that complement conventional instrumental analysis. Beyond detection, they serve as functional readouts of microbial stress responses, bridging chemical exposure and biological effect. The authors outlined a roadmap for developing robust PFAS-specific bioreporters through sensitive promoter-reporter fusions and sensor strain optimization. Therefore, developing robust biological sensing technologies may ultimately support more adaptive monitoring and management of engineered water and wastewater systems.

In addition, at the molecular scale, Yu investigated the differential responses of the marine ciliate Euplotes vannus to three structurally similar tetracyclines, including chlortetracycline (CTC), doxycycline (DOX), and tetracycline (TCH). Through comparative transcriptomics and biochemical assays, they uncovered compound-specific defense strategies: CTC triggered global metabolic reprogramming accelerating lipid degradation to fuel cytochrome P450-mediated detoxification; DOX induced endoplasmic reticulum stress requiring chaperone upregulation; while TCH caused translational arrest and energy downregulation. Critically, the study revealed that structural similarity does not guarantee mechanistic similarity, highlighting the need for compound-specific risk assessment. Meanwhile, this contribution also directly illustrates the concept of metabolic reprogramming. Under environmental stress, microorganisms cannot allocate unlimited resources simultaneously to growth, reproduction, detoxification, cellular repair, and stress defense. Instead, energy and metabolic resources must be redistributed according to environmental conditions. Molecular defense strategies may therefore enhance survival while imposing physiological costs. Such trade-offs are highly relevant to engineered microbial ecosystems, where chronic exposure to emerging contaminants may alter not only microbial diversity but also metabolic efficiency and ecosystem functioning.

Taken together, the four contributions form a multi-scale framework for understanding microbial responses to emerging contaminants. At the molecular level, microorganisms activate defense mechanisms and redistribute metabolic resources to survive environmental stress. At the genetic level, MGEs and horizontal gene transfer may accelerate adaptation and facilitate the dissemination of functional traits (Raju et al., 2025). At the community level, ecological interactions and trophic organization influence microbial assembly, functional stability, and resilience. Finally, biological sensing technologies provide opportunities to monitor the interface between chemical exposure and microbial response. These interconnected processes suggest that microbial adaptation should not be considered as a linear response. Instead, emerging contaminant stress can initiate feedbacks across multiple biological scales. Molecular responses may alter cellular fitness; differences in fitness can reshape community composition; community restructuring can influence functional performance; and environmental conditions can further select for specific metabolic or genetic traits. Understanding these feedbacks will be essential for predicting the long-term behavior of engineered microbiomes.

Future research should therefore move toward an integrated systems-level perspective. Multi-omics approaches can help connect microbial community composition with gene expression, metabolic activity, and ecosystem function. Single-cell technologies may further reveal the heterogeneity of microbial responses that is often masked by bulk community analyses. At the same time, improved biosensors and real-time biological monitoring could provide dynamic information on microbial stress and contaminant bioavailability. Integrating these approaches with ecological theory and process engineering may enable the development of predictive models for microbial resilience.

Overall, the contributions in this Research Topic demonstrate that the study of emerging contaminants should extend beyond pollutant occurrence and removal. Emerging contaminants interact continuously with microbial systems, influencing molecular physiology, metabolic allocation, genetic exchange, community organization, and ecosystem stability. The central challenge for future environmental microbiology will be to translate this mechanistic understanding into strategies for engineering more resilient, efficient, and safe microbial ecosystems.

By bringing together perspectives from molecular biology, microbial ecology, environmental biotechnology, genetic mobility, and biosensing, this Research Topic provides a multi-scale view of microbial adaptation under emerging contaminant stress. Future progress will depend on linking these scales, from cellular defense and metabolic reprogramming to community assembly and ecosystem resilience. Such integration will be essential for designing the next generation of engineered water and wastewater treatment systems capable of responding to increasingly complex environmental challenges.
